# Supplementary material for: Identification of photoperiod-induced specific miRNAs in the adrenal glands of Sunite sheep (Ovis aries)
Source: Front Vet Sci. 2022 Jul 22;9:888207. doi: 10.3389/fvets.2022.888207 (PMC9354845; doi:10.3389/fvets.2022.888207)
Supplement: Supplementary file 1 [file Data_Sheet_1.ZIP › Supplementary file/miRDeep2 file.zip!/miRDeep2 file/Know_Structure/LP42A2/oar-mir-29b-1.pdf]

| | |
|---|---|
| miRBase precursor | : oar-mir-29b-1 |
| Total read count | : 12799 |
| oar-miR-29b read count | : 11857 |
| remaining reads | : 942 |

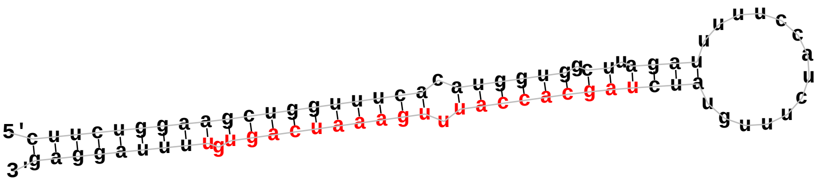

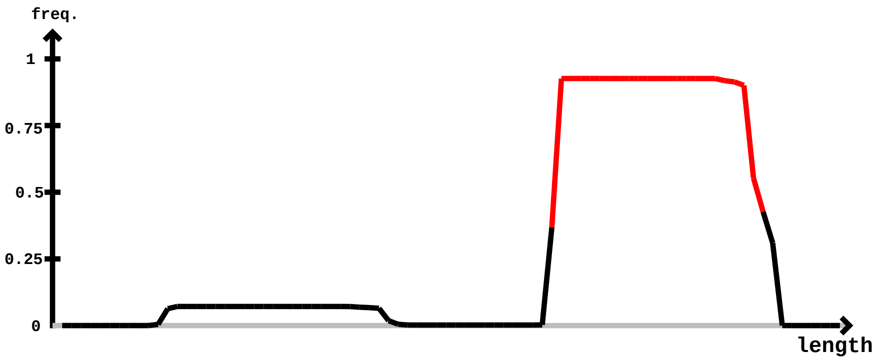


oar-miR-29b

| 5'- cuucuggaagcugguuucacaugguggcuuagauuuuuccaucuuuguaucuagcaccauuugaaaucaguguuuuaggag -3' | exp | | |
|---|---|---|---|
| (((((((((((((((((((.((((((.((.((((..............)))))))))))).)))))))))).))))))))) | reads | mm | sample |
| .........gcugguuucacaugguggcu.................................................... | 4 | 0 | seq |
| .........gcugguuucacaugguggcuu................................................... | 7 | 0 | seq |
| .........gcugguuucacaugguggcuua.................................................. | 4 | 0 | seq |
| .........gcugguuucacaugguggcuuag................................................. | 5 | 0 | seq |
| .........gcugguuucacaugguggcuuaga................................................ | 36 | 0 | seq |
| .........gcugguuucacaugguggcuuagau............................................... | 1 | 0 | seq |
| ..........cugguuucacaugguggcuu................................................... | 24 | 0 | seq |
| ..........cugguuucacaugguggcuua.................................................. | 15 | 0 | seq |
| ..........cugguuucacaugguggcuuag................................................. | 25 | 0 | seq |
| ..........cugguuucacaugguggcuuaga................................................ | 535 | 0 | seq |
| ..........cugguuucacaugguggcuuagau............................................... | 133 | 0 | seq |
| ..........cugguuucacaugguggcuuagauu.............................................. | 19 | 0 | seq |
| ..........cugguuucacaugguggcuuagauuu............................................. | 2 | 0 | seq |
| ...........ugguuucacaugguggcuu................................................... | 2 | 0 | seq |
| ...........ugguuucacaugguggcuuag................................................. | 3 | 0 | seq |
| ...........ugguuucacaugguggcuuaga................................................ | 48 | 0 | seq |
| ...........ugguuucacaugguggcuuagau............................................... | 39 | 0 | seq |
| ...........ugguuucacaugguggcuuagauu.............................................. | 15 | 0 | seq |
| .................................uuuuuccaucuuuguau............................... | 2 | 0 | seq |
| .................................uuuuuccaucuuuguauA.............................. | 1 | 1 | seq |
| .................................uuuuuccaucuuuguauU.............................. | 2 | 1 | seq |
| .................................uuuuuccaucuuuguauc.............................. | 18 | 0 | seq |
| .................................uuuuuccaucuuuguaucC............................. | 1 | 1 | seq |
| ..................................uuuuccaucuuuguaucuagca......................... | 1 | 0 | seq |
| .................................................ucuagcaccauuugaaauc............. | 1 | 0 | seq |
| .................................................ucuagcaccauuugaaaucagu.......... | 5 | 0 | seq |
| ..................................................cuaCcaccauuugaaauc............. | 1 | 1 | seq |
| ..................................................cuagcaccauuugaaauc............. | 47 | 0 | seq |
| ..................................................cuagcaccauuugaaauca............ | 14 | 0 | seq |
| ..................................................cuagcaccauuugaaaucag........... | 11 | 0 | seq |
| ..................................................cuagcaccauuugaaaucagu.......... | 2332 | 0 | seq |
| ..................................................cuagcaccauuugaaaucGgu.......... | 26 | 1 | seq |
| ..................................................cuagcaccauuugaaaucagug......... | 1238 | 0 | seq |
| ..................................................cuagcaccauuugaaaucagugu........ | 1009 | 0 | seq |
| ..................................................cuagcaccauuugaaaucaguguu....... | 5 | 0 | seq |

oar-miR-29b

```
cuucuggaagcugguuucacaugguggcuuagauuuuuccaucuuuguaucuagcaccauuugaaaucaguguuuuaggag
...................................................uagcaccauuugaaauc.............    50     0     seq
...................................................uagcaccauuugaaauU.............    5      1     seq
...................................................uaUcaccauuugaaauc.............    1      1     seq
...................................................uagcaccauuugaaauca............    25     0     seq
...................................................uagcaccauuugaaaucG............    17     1     seq
...................................................uagcaccauuugaaaucag...........    29     0     seq
...................................................uagcaccauuugaaaucGg...........    126    1     seq
...................................................uagcaccauuugaaaucGgu..........    756    1     seq
...................................................uagcaccauuugaaaucagu..........    1323   0     seq
...................................................uagcaccauuugaaaucagug.........    387    0     seq
...................................................uagcaccauuugaaaucagugu........    488    0     seq
...................................................uagcaccauuugaaaucaguguu.......    3954   0     seq
...................................................uagcaccauuugaaaucaguguuu......    2      0     seq
....................................................agcaccauuugaaaucGgu..........    1      1     seq
....................................................agcaccauuugaaaucagu..........    1      0     seq
....................................................agcaccauuugaaaucagugu........    1      0     seq
....................................................agcaccauuugaaaucaguguu.......    2      0     seq
```
